# Estimating the Population Benefits of Blood Pressure Lowering: A Wide‐Angled Mendelian Randomization Study in UK Biobank

**DOI:** 10.1161/JAHA.121.021098

**Published:** 2021-08-28

**Authors:** Hannah Higgins, Amy M. Mason, Susanna C. Larsson, Dipender Gill, Claudia Langenberg, Stephen Burgess

**Affiliations:** ^1^ Department of Public Health and Primary Care University of Cambridge United Kingdom; ^2^ Department of Surgical Sciences Uppsala University Uppsala Sweden; ^3^ Unit of Cardiovascular and Nutritional Epidemiology Institute of Environmental Medicine Karolinska Institutet Stockholm Sweden; ^4^ Department of Epidemiology and Biostatistics School of Public Health Imperial College London London United Kingdom; ^5^ Department of Genetics Novo Nordisk Research Centre Oxford Oxford United Kingdom; ^6^ Clinical Pharmacology and Therapeutics Section Institute of Medical and Biomedical Education and Institute for Infection and Immunity St George's, University of London London United Kingdom; ^7^ Clinical Pharmacology Group Pharmacy and Medicines Directorate St George's University Hospitals National Health Service Foundation Trust London United Kingdom; ^8^ Medical Research Council Epidemiology Unit University of Cambridge United Kingdom; ^9^ Computational Medicine Berlin Institute of Health Charité Universitätsmedizin Berlin Germany; ^10^ Medical Research Council Biostatistics Unit University of Cambridge Cambridge United Kingdom

**Keywords:** cardiovascular disease, genetic epidemiology, high blood pressure, hypertension, Mendelian randomization, Epidemiology, Cardiovascular Disease, Risk Factors, High Blood Pressure

## Abstract

**Background:**

The causal relevance of elevated blood pressure for several cardiovascular diseases (CVDs) is uncertain, as is the population impact of blood pressure lowering. This study systematically assesses evidence of causality for various CVDs in a 2‐sample Mendelian randomization framework, and estimates the potential reduction in the prevalence of these diseases attributable to long‐term population shifts in the distribution of systolic blood pressure (SBP).

**Methods and Results:**

We investigated associations of genetically predicted SBP as predicted by 256 genetic variants with 21 CVDs in UK Biobank, a population‐based cohort of UK residents. The sample consisted of 376 703 participants of European ancestry, aged 40 to 69 years at recruitment. Genetically predicted SBP was positively associated with 14 of the outcomes (*P*<0.002), including dilated cardiomyopathy, endocarditis, peripheral vascular disease, and rheumatic heart disease. Using genetic variation to estimate the long‐term impact of blood pressure lowering on disease in a middle‐aged to early late‐aged UK‐based population, population reductions in SBP were predicted to result in an overall 16.9% (95% CI, 12.2%–21.3%) decrease in morbidity for a 5–mm Hg decrease from a population mean of 137.7 mm Hg, 30.8% (95% CI, 22.8%–38.0%) decrease for a 10–mm Hg decrease, and 56.2% (95% CI, 43.7%–65.9%) decrease for a 22.7–mm Hg decrease in SBP (22.7 mm Hg represents a shift from the current mean SBP to 115 mm Hg).

**Conclusions:**

Risk of many CVDs is influenced by long‐term differences in SBP. The burden of a broad range of CVDs could be substantially reduced by long‐term population‐wide reductions in the distribution of blood pressure.

High blood pressure has severe, costly consequences largely through increased cardiovascular disease (CVD) risk.^1^ For many CVDs, a causal relationship with blood pressure that is reversible through treatment has been demonstrated in randomized controlled trials (RCTs).^2^ However, for diseases such as dilated cardiomyopathy, endocarditis, peripheral vascular disease, and aortic valve stenosis, RCT evidence demonstrating a causal effect of blood pressure lowering is lacking. Despite this, these outcomes have been used to estimate the population impact of increased blood pressure.^3^ In addition, quantitative evidence for the benefit of reducing blood pressure has not been assessed for several CVD outcomes in a primary prevention setting.

In the absence of RCT evidence, Mendelian randomization (MR) can circumvent several limitations of observational epidemiology, allowing unconfounded inferences from observational data.^4^ MR uses selected genetic variants related to an exposure to provide evidence supporting a causal hypothesis. The independent segregation of alleles at conception means that genetically defined subgroups of the population with increased or decreased average blood pressure levels should not differ systematically with respect to confounding variables, creating a natural experiment analogous to an RCT. Life‐long average differences in the exposure between subgroups compared in an MR analysis provide evidence on the potential impact of long‐term interventions on the exposure, in contrast to the short‐term interventions usually evaluated by RCTs.^5^

Herein, we use MR to assess evidence for causality between systolic blood pressure (SBP) and a broad range of CVDs in UK Biobank, a population‐based cohort of UK residents. We then use these estimates to predict the potential reduction in CVD burden in the UK population attributable to distributional shifts in SBP. We focus on SBP as a measure of blood pressure because it is a better predictor of health outcomes than diastolic blood pressure.^1^ However, as the genetic variants used in this investigation are associated with both SBP and diastolic blood pressure, estimates relate generally to blood pressure lowering and are not specific to SBP.

## Methods

Summarized genetic data used in this investigation have been made publicly available and can be accessed at https://doi.org/10.6084/m9.figshare.14417594.v1. The UK Biobank study was approved by the UK's North West Multi‐Centre Research Ethics Committee. All participants provided written informed consent.

We performed 2‐sample MR analyses using summarized data (Figure S1). Genetic associations with blood pressure were obtained in an analysis of 299 024 European ancestry participants from the International Consortium for Blood Pressure, which excluded UK Biobank participants.^6^ Genetic associations with 21 CVDs were estimated in 376 703 European ancestry participants from UK Biobank by logistic regression with adjustment for age, sex, and 10 genomic principal components (Table S1). Derivation of the analytic subset followed quality control steps described previously^7^: after filtering genetic variants (call rate ≥99%, information score >0.9, and Hardy‐Weinberg equilibrium *P*≥10^−5^) and participants (removal of genetic sex mismatches), we excluded participants having non‐European ancestries (self‐report or inferred by genetics) or excess heterozygosity (>3 SDs from the mean), and included only one of each set of related participants (third‐degree relatives or closer).

As genetic instruments, we selected 256 variants previously associated with blood pressure at a genome‐wide level of significance in the International Consortium for Blood Pressure data set, excluding UK Biobank participants (Table S2). As the International Consortium for Blood Pressure and UK Biobank samples do not overlap, bias attributable to winner's curse is avoided. The variants explained 2.1% of variance in SBP in International Consortium for Blood Pressure, corresponding to an F‐statistic of 23.1. Associations between a weighted genetic risk score and potential confounders (sex, age, body mass index [BMI], smoking status, low‐density lipoprotein [LDL] cholesterol, alcohol drinker status, glycated hemoglobin, history of diabetes mellitus at recruitment, and physical activity) were assessed in UK Biobank.

### Statistical analysis

MR estimates for the casual effect of SBP on the odds of 21 CVD outcomes were obtained using the inverse variance weighted (IVW) method.^4^ Estimates are odds ratios per 10–mm Hg increase in genetically predicted SBP. Sensitivity analyses were performed using the MR‐Egger, weighted median, and Mendelian Randomization‐Pleiotropy Residual Sum and Outlier (MR‐PRESSO) methods.^4^

The plausibility of estimates derived from MR having a causal interpretation relies on genetic variants satisfying 3 assumptions: the genetic variant is associated with the exposure of interest (the relevance assumption); the genetic variant can only influence the outcome through its effect on the exposure (the exclusion restriction assumption); and the genetic variant is not associated with the outcome via a confounding pathway (the exchangeability assumption). The IVW method assumes that all genetic variants satisfy these assumptions, or that the average pleiotropic effect across genetic variants is zero.^4^ The additional methods provide reliable inferences when some genetic variants violate these assumptions, allowing investigation of the robustness of results. The MR‐Egger method relaxes the IVW assumption that the average pleiotropic effect is zero, allowing for directional pleiotropy when the pleiotropic effects of the genetic variants on the outcome are not correlated with their associations with the exposure. The weighted median method takes a median (instead of a weighted mean) of the variant‐specific estimates, providing an estimate robust to outlying variants. In the MR‐PRESSO method, variants with heterogeneous estimates (which may be pleiotropic variants) are excluded from the analysis, and the IVW method is subsequently performed, omitting such variants.

Outcomes with consistent evidence for causality (*P*<0.05/21=0.002 in either the IVW or the MR‐PRESSO method and concordant direction of estimates across all methods) were used to estimate the change in disease burden that would occur under interventions in the distribution of SBP. Assuming a linear model, we estimated the population impact fraction,^3^ representing the relative reduction in disease risk if mean SBP was set to a given value for all individuals in the population. We also estimated the absolute reduction in events from a population shift in the distribution of SBP using disease prevalence estimates from surveys relevant to a middle‐aged to early late‐aged UK‐based population (Table S3).

All analyses were performed in R version 3.6.3 (“Holding the Windsock”).

## Results

SBP was approximately normally distributed, with a mean of 137.7 mm Hg (SD, 18.6 mm Hg) (Figure S2). There was no association between the genetic risk score and age, sex, smoking, physical activity, glycated hemoglobin, or alcohol consumption (Table). The genetic risk score was associated with SBP, BMI, LDL cholesterol, and history of diabetes mellitus, but the absolute magnitude of associations other than with SBP was small (mean difference between top versus bottom 50%: 4.1 mm Hg for SBP, −0.1 kg/m^2^ for BMI, −0.04 mmol/L for LDL cholesterol, and 0.4% for prevalence of diabetes mellitus history). Indeed, any pleiotropic influence of BMI or LDL cholesterol would generally result in underestimation of the effect of SBP as both BMI and LDL cholesterol increase the risk of most CVDs.

**Figure 1:**
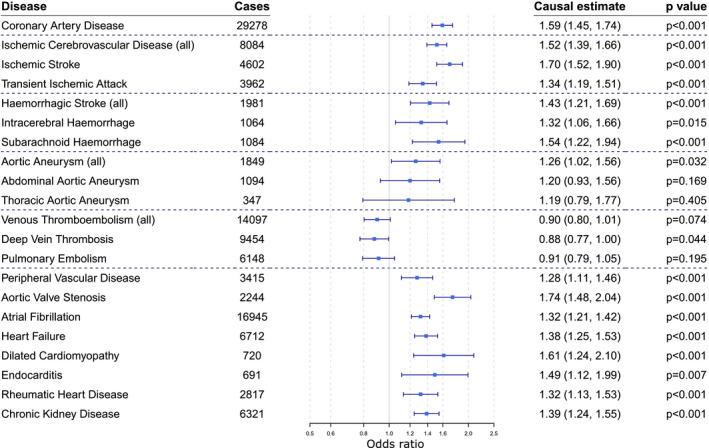
Mendelian randomization estimates (odds ratio with 95% CI per 10–mm Hg increase in genetically predicted systolic blood pressure) from the inverse variance weighted method. To account for multiple testing, *P*<0.002 (0.05/21) is considered as the threshold for statistical significance.

Fourteen outcomes satisfied the criteria of consistent evidence for causality: *P*<0.002 in either the IVW or the MR‐PRESSO method and concordance of estimates across methods (Figure 1 and Table S4). In decreasing order of the IVW estimate, these were: aortic valve stenosis, ischemic stroke, dilated cardiomyopathy, coronary artery disease, subarachnoid hemorrhage, ischemic cerebrovascular disease, endocarditis, hemorrhagic stroke (all), chronic kidney disease, heart failure, transient ischemic attack, atrial fibrillation, rheumatic heart disease, and peripheral vascular disease (Figure S3). Two further outcomes (intracerebral hemorrhage and aortic aneurysm) had a positive IVW estimate at a conventional level of statistical significance (*P*<0.05). Deep vein thrombosis had an inverse IVW estimate at a conventional level of statistical significance (*P*<0.05).

**Figure 2:**
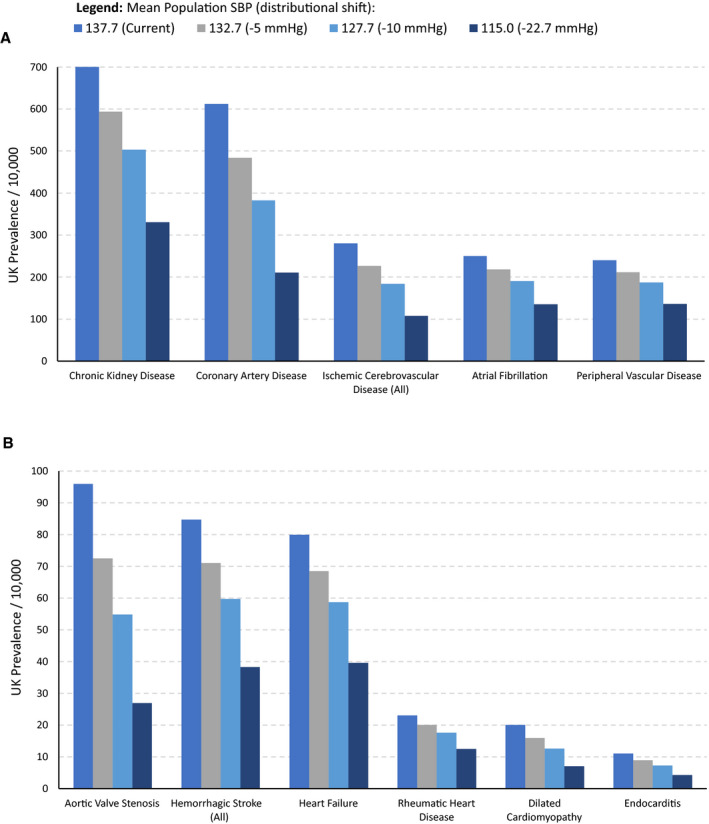
Bar chart showing the prevalence and estimated changes if lifelong systolic blood pressure (SBP) decreased across its distribution by 5, 10, or 22.7 mm Hg (22.7 mm Hg represents a shift from the current mean of 137.7 to 115 mm Hg) separately for each cardiovascular outcome and for high‐prevalence outcomes (current UK prevalence >200 per 10 000) (**A**) and lower‐prevalence outcomes (current UK prevalence <200 per 10 000) (**B**). Ischemic cerebrovascular disease (all) comprises ischemic stroke and transient ischemic attack. Hemorrhagic stroke (all) comprises subarachnoid hemorrhage and intracerebral hemorrhage.

Figure 2 shows the estimated changes in the absolute prevalence of outcomes with consistent evidence for causality resulting from a population shift in the distribution of SBP. Population impact fractions for these outcomes are provided in Table S5. Aggregating across these outcomes, reductions in SBP were predicted to result in an overall 16.9% (95% CI, 12.2%–21.3%) decrease in CVD morbidity for a 5–mm Hg SBP decrease from a population mean of 137.7 mm Hg, 30.8% (95% CI, 22.8%–38.0%) decrease for a 10–mm Hg SBP decrease, and 56.2% (95% CI, 43.7%–65.9%) decrease for a 22.7–mm Hg SBP decrease. The value of 22.7 mm Hg represents a shift from the current mean SBP in the population to 115 mm Hg, a value that has been proposed as a theoretical minimum risk target.^3^

## Discussion

Although for many CVDs, the causal effect of blood pressure lowering has been demonstrated convincingly in RCTs, several outcomes (dilated cardiomyopathy, endocarditis, peripheral vascular disease, and rheumatic heart disease) had previously only been shown to be associated with SBP in observational studies.^1^, ^3^ Our analysis adds evidential weight to blood pressure as a causal risk factor for these outcomes and to their inclusion in other SBP population impact studies.

Although most of the outcomes associated with genetically predicted SBP are chronic diseases, some have infectious origins (eg, endocarditis and rheumatic heart disease). Elevated SBP may therefore increase susceptibility to, or damage from, infection. A notable finding was the inverse association between genetically predicted SBP and deep vein thrombosis. Although this association did not achieve statistical significance after accounting for multiple testing, some evidence for an inverse association between SBP and venous thromboembolism has been found in previous observational studies.^8^

The MR estimates obtained herein were generally larger than those from RCTs and conventional observational analyses. For example, the MR estimate for the risk of ischemic stroke per 10–mm Hg increase in SBP was an odds ratio of 1.70 (95% CI, 1.52–1.90). This contrasts with relative risk estimates of 1.37 (95% CI, 1.30–1.47) from a recent meta‐analysis of RCTs,^2^ and 1.53 (95% CI, 1.49–1.56) for stroke in 60‐ to 69‐year‐old people in the observational analysis of the Prospective Studies Collaboration.^9^ Although some difference between estimates is expected as MR estimates represent the lifelong impact of elevated blood pressure, whereas RCTs vary blood pressure for a shorter period, other factors, such as trial setting and inclusion criteria, may also contribute to differences.

This study has many strengths, but also limitations. The large sample size and wide range of outcomes enable systematic cross‐comparisons of unconfounded estimates in a single cohort. This is important from a public health perspective when comparing the impact of the same genetic change on different diseases. The genome‐wide genotypic data available from the UK Biobank cohort absolved any need to use proxy variants in the instrument. However, the results should be interpreted in the context of several limitations.

First, the UK Biobank cohort is somewhat unrepresentative of the UK population and experiences a “healthy volunteer” selection bias.^10^ Analyses were conducted in participants of European descent to avoid population stratification. Estimates may therefore not be fully relevant for the whole UK population. Whether the findings are applicable to other races/ethnicities warrants investigation, particularly because hypertension disproportionately affects Black African and Caribbean ancestry racial/ethnic groups in both the UK and abroad.^11^ Given large global disparities in hypertension prevalence, generalizability of the public health modeling beyond the United Kingdom may be limited.

The MR approach is underpinned by assumptions that cannot be completely empirically validated. However, the genetic risk score for SBP was not strongly associated with major confounders, and estimates were generally similar across robust methods.

Although the principles of MR seek to emulate an RCT, the approach differs fundamentally in certain aspects, which are relevant when using MR in public health modeling. First, the results presented herein reflect lifelong differences in SBP relating to genetic variants that are determined at conception. The reversibility of these long‐term effects is unknown; however, reversibility is assumed in the population impact fraction calculations. Although SBP‐associated risks have demonstrated reversibility in RCTs for most cardiovascular outcomes, it is unknown whether this applies to the full spectrum of outcomes studied herein. There may be no existing intervention applicable to a mature cohort that can imitate the genetic effect, and if such an intervention does exist, the time lag or age at treatment onset required to produce the predicted effects is unknown. For context, antihypertensive drug treatment reduced SBP by 8.3 mm Hg in patients aged 30 to 49 years, 10.7 mm Hg in patients aged 60 to 79 years, and 9.4 mm Hg in patients aged >80 years in a meta‐analysis of RCTs of patients with isolated systolic hypertension (SBP >160 mm Hg).^12^ Population‐wide shifts in the distribution of SBP using nonclinical interventions are generally much smaller in magnitude and occur over much larger time frames: for example, the UK population's average SBP has decreased by ≈3 mm Hg between 2003 and 2017.^11^

There are several limitations to use of the population impact factors. First, our results are tailored to a middle‐aged to early late‐aged UK‐based population. We focus on this group as the estimates obtained from UK Biobank are most relevant to this population. We did not attempt subgroup or interaction analyses to investigate the impact of blood pressure lowering in different subgroups of the population, or in groups with comorbidities. The population impact fraction measure used herein assumes independence of effects on different outcomes; in reality, the outcomes considered are frequently consequential or coincident in patients. However, although dependence between the outcomes would inflate CIs for the public health modeling estimates, it would not affect the estimates themselves as the expected value of the sum of estimates is equal to the sum of the expectations of the estimates, even if the estimates are correlated. Finally, these analyses assume linearity of effects. Estimates are likely to be reliable for shifts in SBP of similar magnitude to the genetic associations with SBP, which are around 8 to 10 mm Hg.^4^ The appropriateness of extrapolation to larger changes in SBP cannot be assessed in this current study.

The evidence presented herein suggests that incidence of CVDs is influenced by long‐term differences in the distribution of SBP, even in a population‐based sample. Therefore, confining blood pressure–lowering interventions to older age groups and individuals over a certain risk threshold will likely only partially address the totality of disease burden. Although these estimates constitute a modeling exercise and not an intervention analysis, they do provide evidence to support a life course approach to lowering population SBP. Several other publications support this stance. A recent MR study found evidence indicating a linear relationship of genetically predicted SBP with coronary artery disease, further supporting the conclusion that even individuals with SBP in the normal range can benefit from public health interventions achieving persistent SBP reduction for the primary prevention of CVD.^13^ In another MR investigation, naturally occurring random allocation to higher SBP instrumented by multiple common genetic variants was associated with a significantly faster increase in SBP with age, and this increased exposure to elevated blood pressure had a cumulative detrimental effect on the risk of coronary heart disease greater than that observed in RCTs.^14^ Further work has used the observation that some genetic predictors of blood pressure more strongly predict midlife blood pressure, whereas others more strongly predict later‐life blood pressure. Genetically predicted midlife blood pressure was shown to be independently associated with coronary artery disease risk when conditioning on later‐life blood pressure, suggesting that these represent distinct risk factors.^15^ Interventions that target the determinants of high blood pressure at early stages in the life course, and earlier identification and treatment of those with high blood pressure, are therefore more likely to yield the large effect sizes observed herein than those that only target people already in a later stage of life. This provides an important opportunity to reduce the inequality in health outcomes along the socioeconomic gradient. Interventions that may replicate the lifelong reduction in exposure to elevated SBP evaluated herein include nonpharmacologic interventions, such as increased physical activity, weight control, and sodium reductions, as well as community‐wide programs, such as consumer awareness campaigns and industry collaboration for food reformulation.

In conclusion, reducing SBP by 10 mm Hg could reduce the overall burden of a broad range of CVDs by around 30%. These findings contribute to an ever‐expanding body of evidence advocating targeted and population‐based strategies for management of high blood pressure across the life course.

## Sources of Funding

Dr Mason is funded by the National Institute for Health Research (Cambridge Biomedical Research Centre at the Cambridge University Hospitals National Health Service Foundation Trust) and by a European Council Innovative Medicines Initiative (BigData@Heart). Dr Larsson has received grants from the Swedish Research Council for Health, Working Life and Welfare (Forte; grant No. 2018‐00123), the Swedish Research Council (Vetenskapsrådet; grant No. 2019‐00977), and the Swedish Heart‐Lung Foundation (Hjärt‐Lungfonden; grant No. 20190247). Dr Burgess is supported by Sir Henry Dale Fellowship jointly funded by the Wellcome Trust and the Royal Society (204623/Z/16/Z). Dr Gill is supported by the Wellcome Trust 4i Programme (203928/Z/16/Z) and British Heart Foundation Centre of Research Excellence (RE/18/4/34215) at Imperial College London, and a National Institute for Health Research Clinical Lectureship at St. George's, University of London (CL‐2020‐16‐001). Dr Langenberg is funded by the Medical Research Council. This research was supported by the National Institute for Health Research Cambridge Biomedical Research Centre (BRC‐1215‐20014).

## Disclosures

Dr Gill is employed part‐time by Novo Nordisk outside the submitted work. Hannah Higgins is employed full‐time by Public Health England outside the submitted work. The remaining authors have no disclosures to report.

**Table 1:** Characteristics of Participants in the Analytic Subset of the UK Biobank Study

Characteristics	Overall	Genetic risk score percentile		*P* value
		Lower 50%	Upper 50%	
No. of participants	367 703	183 822	183 821	…
Age at survey, mean (SD), y	57.2 (8.0)	57.2 (8.0)	57.2 (8.0)	0.26
Women, n (%)	198 902 (54.1)	99 564 (54.2)	99 307 (54.0)	0.39
Body mass index, mean (SD), kg/m^2^	27.4 (4.8)	27.4 (4.8)	27.3 (4.7)	<0.001
Low‐density lipoprotein, mean (SD), mmol/L	3.57 (0.87)	3.59 (0.87)	3.55 (0.87)	<0.001
HbA1c, mean (SD), mmol/mol	35.9 (6.4)	35.9 (6.4)	35.9 (6.5)	0.10
History of diabetes mellitus at recruitment, n (%)	16 927 (4.6)	8082 (4.4)	8843 (4.8)	<0.001
Physical activity, mean (SD), MET min/wk	2660 (2710)	2650 (2710)	2669 (2709)	0.06
Deaths, n (%)	8033 (2.2)	3925 (2.1)	4105 (2.2)	0.04
Cardiovascular events, n (%)	7145 (1.9)	3291 (1.8)	3853 (2.1)	<0.001
Cardiovascular events (fatal), n (%)	1422 (0.4)	682 (0.4)	740 (0.4)	0.12
Cardiovascular events (nonfatal), n (%)	5723 (1.6)	2609 (1.4)	3113 (1.7)	<0.001
Cerebrovascular events (fatal), n (%)	2714 (0.7)	1217 (0.7)	1497 (0.8)	<0.001
Current smokers, n (%)	37 866 (10.3)	19 099 (10.4)	18 763 (10.2)	0.07
Current alcohol drinkers, n (%)	342 797 (93.4)	171 486 (93.4)	171 260 (93.3)	0.25
Participants taking antihypertensives, n (%)	74 556 (20.4)	29 841 (16.4)	44 702 (24.5)	<0.001
Systolic blood pressure, mean (SD), mm Hg	137.7 (18.6)	135.6 (18.2)	139.7 (18.8)	<0.001
Diastolic blood pressure, mean (SD), mm Hg	82.0 (10.1)	81.1 (10.0)	82.8 (10.2)	<0.001

Genetic risk score was calculated from 256 variants previously associated with a blood pressure trait at *P*<5×10^−8^ in data from the International Consortium for Blood Pressure, excluding UK Biobank participants. Physical activity is measured in MET minutes per week, and is taken from Data Field 22 040. HbA1c indicates glycated hemoglobin; and MET, metabolic equivalent task.

## Supporting information

Tables S1–S5
Figures S1–S3
