# Supplementary material for: Estimating the Population Benefits of Blood Pressure Lowering: A Wide‐Angled Mendelian Randomization Study in UK Biobank
Source: J Am Heart Assoc. 2021 Aug 28;10(17):e021098. doi: 10.1161/JAHA.121.021098 (PMC8649307; doi:10.1161/JAHA.121.021098)
Supplement: Supplementary file 1 — Tables S1–S5 Figures S1–S3 [file JAH3-10-e021098-s001.pdf]

Supplemental Material

Table S1. Summary of disease outcomes considered, definitions, and sources of information within UK Biobank

Outcome name	ICD-9 Diagnosis or death	ICD-10 Diagnosis or death	OPCS procedure code	Self-report[a]
**Coronary Artery Disease**	410.X, 411.X, 412.X, 414.0, 414.8, 414.9	I21.X, I22.X, I23.X, I24.X, I25.1, I25.2, I25.5, I25.6, I25.8, I25.9	K40.X, K41.X, K42.X, K43.X, K44.X, K45.X, K46.X, K49.X, K50.1, K50.2, K50.4, K75.X	Non-cancer illness code (20002), Surgical operation code (20004), Health condition diagnosed by doctor (6150)
**Ischemic Cerebrovascular Disease (all)**	434.X, 435.X, 436.X	G45.X, I63.X, I64.X		Non-cancer illness code (20002)
**Ischemic Stroke**	433.X, 434.X	I63.X, I64.X		Non-cancer illness code (20002)
**Transient Ischemic Attack**	435.X	G45.X		Non-cancer illness code (20002)
**Hemorrhagic Stroke (all)**	430.X, 431.X	I60.X, I61.X		Non-cancer illness code (20002)
**Intracerebral Hemorrhage**	431.X	I61.X		Non-cancer illness code (20002)
**Subarachnoid Hemorrhage**	430.X	I60.X		Non-cancer illness code (20002)
**Aortic Aneurysm (all)**	441.X	I71.X	L19.4, L19.5	Non-cancer illness code (20002), Surgical operation code (20004)
**Abdominal Aortic Aneurysm**	441.3, 441.4	I71.3, I71.4	L19.4, L19.5	Non-cancer illness code (20002)
**Thoracic Aortic Aneurysm**	441.1, 441.2	I71.1, I71.2		Non-cancer illness code (20002)
**Venous Thromboembolism (all)**	415.1, 451.1, 452.X, 453.0, 453.4, 453.9,	I26.X, I80.1, I80.2, I81.X, I82.0	L90.2	Non-cancer illness code (20002), Health condition diagnosed by doctor (6152)
**Deep Vein Thrombosis**	451.1	I80.2	L90.2	Non-cancer illness code (20002), Health condition diagnosed by doctor (6152)
**Pulmonary Embolism**	415.1	I26.X		Non-cancer illness code (20002), Health condition diagnosed by doctor (6152)

Outcome name	ICD-9 Diagnosis or death	ICD-10 Diagnosis or death	OPCS procedure code	Self-report*
**Peripheral Vascular Disease**	443.8, 443.9	I73.8, I73.9		Non-cancer illness code (20002)
**Aortic Valve Stenosis**	424.1	I35.0, I35.2		Non-cancer illness code (20002)
**Atrial Fibrillation**	427.3	I48		Non-cancer illness code (20002)
**Heart Failure**	402.01, 402.11, 402.91, 404.01, 404.11, 404.91, 404.03, 404.13, 404.93, 428.X	I11.0, I13.0, I13.2, I50.X		Non-cancer illness code (20002)
**Dilated Cardiomyopathy**		I42.0		
**Endocarditis**	391.1, 421.0, 421.9, 421.1	I33.X, I38, I39.8, I01.1		
**Rheumatic Heart Disease**	391.X, 397.9, 398.0, 394.1, 398.90, 397.1	I01.X, I02.0, I05.X, I06.X, I07.X, I08.X, I09.X		
**Chronic Kidney Disease**	585.X	N18.X		Non-cancer illness code (20002)

Note that .X means that all sub-codes are matched.
Abbreviations: ICD, International Classification of Disease; OPCS, Office of Population Censuses and Surveys Classification of Surgical Operations and Procedures.
*Health condition diagnosed by doctor (6150/6152) and Medication for health condition (6177) were self-reported from touchscreen; Non-cancer illness code (20002) and Surgical operation code (20004) were self-reported from interview with trained nurse.

Table S2. Details of the 256 single nucleotide polymorphisms (SNPs) from the International Consortium for Blood Pressure (ICBP) genome-wide association study (GWAS) included in the genetic instrument. β_{xj} corresponds to the variant-SBP association estimates (beta-coefficients in mmHg units). These variants are taken from Supplementary Table 24 of Evangelou et al, Nat Genet 2018, and represent variants that were published prior to the inclusion of UK Biobank in the ICBP.

SNP	Chromosome number	Chromosome position	Effect allele	Other allele	Effect allele frequency	R-squared	F-statistic	β_{xj}	β_{xj} Standard error	β_{xj} P-value
rs10057188	5	77837789	A	G	0.4516	5.0E-05	5.5E-02	-1.9E-01	4.8E-02	4.4E-05
rs10059921	5	87514515	T	G	0.0846	5.8E-05	6.3E-02	-3.7E-01	9.2E-02	4.9E-05
rs10077885	5	114390121	A	C	0.498	8.2E-05	8.9E-02	-2.5E-01	4.8E-02	3.5E-07
rs10078021	5	75038431	G	T	0.3744	4.8E-06	5.3E-03	6.2E-02	4.9E-02	2.1E-01
rs10224002	7	151415041	G	A	0.2814	6.1E-05	6.7E-02	2.4E-01	5.3E-02	6.0E-06
rs1036477	15	48914926	G	A	0.1044	5.8E-05	6.3E-02	-3.4E-01	7.4E-02	4.1E-06
rs10418305	19	15278808	G	C	0.897	3.0E-06	3.3E-03	7.8E-02	7.5E-02	3.0E-01
rs1055144	7	25871109	T	C	0.1925	9.7E-06	1.1E-02	1.1E-01	5.8E-02	6.3E-02
rs1060105	12	123806219	T	C	0.2021	8.8E-06	9.6E-03	-1.0E-01	5.7E-02	7.9E-02
rs1063281	2	218668732	T	C	0.6045	7.3E-05	8.0E-02	-2.4E-01	4.8E-02	7.1E-07
rs10818775	9	125755571	T	C	0.1236	4.3E-05	4.7E-02	-2.7E-01	7.0E-02	8.7E-05
rs10826995	10	32082658	C	T	0.2787	8.7E-06	9.5E-03	9.0E-02	5.1E-02	8.1E-02
rs10850411	12	115387796	C	T	0.3006	1.0E-04	1.1E-01	-3.0E-01	4.9E-02	8.1E-10
rs10922502	1	89360158	G	A	0.3593	6.4E-05	7.0E-02	2.3E-01	4.8E-02	2.3E-06
rs10943605	6	79655477	A	G	0.4886	4.1E-05	4.5E-02	1.7E-01	4.6E-02	1.6E-04
rs11008355	10	31412561	C	G	0.2369	7.1E-06	7.7E-03	-8.6E-02	5.4E-02	1.1E-01
rs11030119	11	27728102	A	G	0.2944	3.2E-05	3.5E-02	-1.7E-01	5.1E-02	8.5E-04
rs110419	11	8252853	G	A	0.5071	8.8E-06	9.6E-03	-8.1E-02	4.7E-02	8.5E-02
rs11067763	12	116198341	G	A	0.1031	1.8E-05	2.0E-02	-1.9E-01	7.5E-02	1.1E-02
rs111245230	9	113169775	C	T	0.0338	8.4E-05	9.2E-02	6.9E-01	1.3E-01	1.0E-07
rs11128722	3	14958126	A	G	0.5628	8.4E-05	9.1E-02	-2.5E-01	4.7E-02	8.5E-08
rs11154027	6	121781390	C	T	0.5513	1.1E-07	1.2E-04	9.0E-03	4.8E-02	8.5E-01
rs11191548	10	104846178	C	T	0.0871	4.5E-04	4.9E-01	-1.0E+00	8.2E-02	6.2E-36
rs112184198	10	102604514	A	G	0.1058	1.4E-04	1.6E-01	-5.3E-01	7.6E-02	2.4E-12
rs11229457	11	58207203	T	C	0.2144	7.5E-05	8.2E-02	-2.9E-01	5.6E-02	3.0E-07
rs112557609	1	56576924	A	G	0.3414	6.9E-05	7.5E-02	2.4E-01	4.9E-02	1.1E-06
rs1126464	16	89704365	C	G	0.245	3.4E-05	3.7E-02	1.8E-01	5.7E-02	1.1E-03
rs1126930	12	49399132	C	G	0.0343	5.9E-05	6.4E-02	5.8E-01	1.4E-01	3.9E-05
rs11537751	11	47587452	T	C	0.0521	4.1E-05	4.5E-02	3.9E-01	1.1E-01	2.6E-04
rs11556924	7	129663496	T	C	0.3713	7.8E-05	8.5E-02	-2.5E-01	5.0E-02	5.9E-07
rs11639856	16	24788645	A	T	0.1902	4.3E-05	4.7E-02	-2.3E-01	5.8E-02	6.8E-05
rs11689667	2	85491365	C	T	0.4536	1.9E-05	2.1E-02	-1.2E-01	4.7E-02	1.1E-02
rs11690961	2	46363336	C	A	0.116	2.9E-07	3.1E-04	-2.3E-02	7.2E-02	7.5E-01
rs11701033	21	33788341	G	C	0.1831	4.9E-05	5.3E-02	2.5E-01	5.9E-02	3.2E-05
rs1173771	5	32815028	G	A	0.6024	3.5E-04	3.8E-01	5.2E-01	4.7E-02	6.0E-29
rs11953630	5	157845402	T	C	0.3694	2.5E-04	2.7E-01	-4.5E-01	5.0E-02	5.2E-19
rs11977526	7	46008110	A	G	0.3993	8.3E-05	9.0E-02	-2.5E-01	4.8E-02	1.6E-07
rs12374077	3	185317674	C	G	0.3452	3.5E-05	3.8E-02	1.7E-01	4.8E-02	4.6E-04
rs12405515	1	172357441	T	G	0.5741	3.9E-05	4.2E-02	-1.7E-01	4.6E-02	2.0E-04
rs12408022	1	217718789	T	C	0.2611	6.5E-05	7.1E-02	2.5E-01	5.3E-02	2.4E-06
rs12521868	5	131784393	T	G	0.4156	2.0E-05	2.2E-02	-1.2E-01	4.8E-02	9.7E-03
rs12579720	12	20173764	G	C	0.7621	8.9E-05	9.7E-02	3.0E-01	5.4E-02	2.5E-08
rs12627651	21	44760603	A	G	0.2944	1.9E-04	2.1E-01	4.2E-01	5.3E-02	3.3E-15
rs12628032	22	19967980	T	C	0.3152	4.5E-05	4.9E-02	2.0E-01	5.0E-02	7.7E-05
rs1275988	2	26914364	T	C	0.6055	3.4E-04	3.7E-01	-5.2E-01	4.7E-02	1.8E-28
rs12906962	15	95312071	C	T	0.3293	8.4E-05	9.2E-02	2.7E-01	4.9E-02	6.4E-08
rs12921187	16	4943019	G	T	0.5726	7.7E-05	8.4E-02	2.4E-01	4.6E-02	1.3E-07
rs12940887	17	47402807	T	C	0.3727	8.9E-05	9.7E-02	2.7E-01	4.7E-02	1.4E-08
rs12941318	17	1333598	C	T	0.5018	5.8E-05	6.3E-02	2.1E-01	4.8E-02	1.7E-05
rs12946454	17	43208121	T	A	0.261	1.1E-04	1.2E-01	3.2E-01	5.2E-02	7.3E-10
rs12958173	18	42141977	C	A	0.7	1.4E-04	1.5E-01	-3.5E-01	5.0E-02	1.2E-12
rs13082711	3	27537909	C	T	0.231	7.5E-05	8.2E-02	2.8E-01	5.5E-02	2.8E-07
rs13107325	4	103188709	T	C	0.0722	2.8E-04	3.0E-01	-8.8E-01	9.5E-02	1.8E-20
rs13112725	4	106911742	C	G	0.7682	1.5E-04	1.6E-01	4.0E-01	5.6E-02	1.0E-12

SNP	Chromosome number	Chromosome position	Effect allele	Other allele	Effect allele frequency	R-squared	F-statistic	β_{xj}	β_{xj} Standard error	β_{xj} P-value
rs13139571	4	156645513	A	C	0.2393	8.6E-05	9.3E-02	-3.0E-01	5.4E-02	3.7E-08
rs13205180	6	51832494	T	C	0.4808	2.3E-06	2.5E-03	4.1E-02	4.6E-02	3.8E-01
rs13209747	6	127115454	T	C	0.4465	2.2E-04	2.5E-01	4.1E-01	4.7E-02	9.8E-19
rs1322639	6	169587103	A	G	0.7711	7.5E-06	8.2E-03	8.9E-02	5.6E-02	1.1E-01
rs13238550	7	131059056	A	G	0.3909	3.7E-05	4.0E-02	1.7E-01	4.7E-02	3.3E-04
rs1327235	20	10969030	G	A	0.4631	1.7E-04	1.8E-01	3.5E-01	4.5E-02	3.2E-15
rs13303	3	52558008	C	T	0.5697	1.6E-05	1.7E-02	1.1E-01	4.7E-02	2.1E-02
rs13333226	16	20365654	G	A	0.186	9.2E-05	1.0E-01	-3.4E-01	5.8E-02	7.0E-09
rs13359291	5	122476457	A	G	0.1654	1.2E-04	1.3E-01	4.0E-01	6.2E-02	1.1E-10
rs13420463	2	37517566	G	A	0.2225	7.0E-05	7.7E-02	-2.8E-01	5.6E-02	7.2E-07
rs1344653	2	19730845	G	A	0.5039	3.3E-05	3.6E-02	1.6E-01	4.6E-02	5.8E-04
rs1378942	15	75077367	A	C	0.6563	2.9E-04	3.1E-01	-4.9E-01	4.8E-02	5.0E-24
rs143112823	3	154707967	A	G	0.076	6.1E-05	6.6E-02	-4.0E-01	9.5E-02	2.3E-05
rs1438896	2	145646072	C	T	0.7001	5.8E-05	6.4E-02	-2.3E-01	5.0E-02	5.4E-06
rs1446468	2	164963486	C	T	0.5488	3.2E-04	3.4E-01	4.9E-01	4.7E-02	2.3E-25
rs1449544	8	76591880	C	A	0.4584	6.5E-05	7.1E-02	-2.2E-01	4.6E-02	1.4E-06
rs1458038	4	81164723	T	C	0.2971	4.9E-04	5.4E-01	6.6E-01	5.1E-02	6.1E-39
rs1475130	14	100225144	C	T	0.6571	3.2E-05	3.5E-02	1.6E-01	4.9E-02	7.4E-04
rs147696085	1	51021867	A	G	0.1011	2.7E-06	2.9E-03	-7.4E-02	8.0E-02	3.5E-01
rs1530440	10	63524591	T	C	0.1874	2.0E-04	2.2E-01	-5.0E-01	5.9E-02	2.0E-17
rs1563788	6	43308363	T	C	0.2937	1.0E-04	1.1E-01	3.1E-01	5.0E-02	9.8E-10
rs167479	19	11526765	T	G	0.4732	2.5E-04	2.7E-01	-4.3E-01	5.6E-02	2.5E-14
rs16823124	2	183224127	A	G	0.303	6.1E-05	6.7E-02	2.3E-01	5.0E-02	3.1E-06
rs16851397	3	141134818	G	A	0.0467	8.3E-05	9.1E-02	5.9E-01	1.1E-01	1.9E-07
rs17030613	1	113190807	C	A	0.2191	1.4E-04	1.5E-01	3.9E-01	5.6E-02	2.5E-12
rs17080102	6	151004770	C	G	0.0677	1.7E-04	1.9E-01	-7.2E-01	9.2E-02	4.9E-15
rs17249754	12	90060586	A	G	0.1637	4.7E-04	5.2E-01	-8.0E-01	6.2E-02	2.2E-38
rs17367504	1	11862778	G	A	0.1556	4.3E-04	4.7E-01	-7.8E-01	6.4E-02	4.8E-34
rs17477177	7	106411858	C	T	0.2094	2.8E-04	3.1E-01	5.6E-01	5.6E-02	1.6E-23
rs17608766	17	45013271	C	T	0.1433	2.5E-04	2.7E-01	6.2E-01	6.7E-02	3.9E-20
rs17638167	19	11584818	T	C	0.047	6.6E-05	7.2E-02	-5.2E-01	1.1E-01	1.8E-06
rs1799945	6	26091179	G	C	0.1479	2.0E-04	2.2E-01	5.4E-01	6.6E-02	1.3E-16
rs1813353	10	18707448	C	T	0.3411	2.3E-04	2.5E-01	-4.3E-01	4.8E-02	3.0E-19
rs1876487	2	73114352	C	A	0.7122	7.5E-06	8.2E-03	8.3E-02	5.5E-02	1.3E-01
rs1925153	6	56102780	T	C	0.4401	3.5E-06	3.8E-03	-5.1E-02	4.9E-02	3.0E-01
rs1953126	9	123640500	C	T	0.6453	4.2E-05	4.5E-02	-1.8E-01	4.8E-02	1.2E-04
rs1975487	2	55809054	G	A	0.5124	6.5E-05	7.1E-02	2.2E-01	4.7E-02	2.9E-06
rs2004776	1	230848702	T	C	0.2381	1.1E-04	1.2E-01	3.4E-01	5.4E-02	5.3E-10
rs2014912	4	86715670	C	T	0.8485	1.8E-04	2.0E-01	-5.1E-01	6.4E-02	1.8E-15
rs2034618	15	83799632	T	C	0.2231	2.1E-07	2.3E-04	-1.5E-02	5.5E-02	7.8E-01
rs2071518	8	120435812	T	C	0.2571	4.9E-05	5.3E-02	2.2E-01	5.2E-02	3.1E-05
rs2076328	1	1687482	T	G	0.4748	1.1E-04	1.2E-01	-2.8E-01	5.0E-02	1.4E-08
rs2107595	7	19049388	A	G	0.166	6.8E-05	7.4E-02	3.0E-01	6.2E-02	1.2E-06
rs2157597	1	169201567	T	C	0.3468	8.9E-06	9.7E-03	-8.6E-02	4.9E-02	8.1E-02
rs2240736	17	59485393	T	C	0.7328	1.9E-04	2.1E-01	4.3E-01	5.3E-02	4.5E-16
rs2246438	10	45273079	A	G	0.2779	5.1E-07	5.5E-04	-2.2E-02	5.1E-02	6.7E-01
rs2282978	7	92264410	C	T	0.342	6.0E-06	6.5E-03	-7.0E-02	4.9E-02	1.5E-01
rs2289125	11	89224453	C	A	0.7697	3.6E-05	3.9E-02	1.9E-01	5.8E-02	8.5E-04
rs2291435	4	38387395	T	C	0.5248	7.8E-05	8.6E-02	-2.4E-01	4.6E-02	1.7E-07
rs2304130	19	19789528	G	A	0.0833	3.9E-05	4.2E-02	3.1E-01	8.6E-02	3.6E-04
rs2306374	3	138119952	C	T	0.1619	3.7E-05	4.1E-02	2.3E-01	6.3E-02	3.0E-04
rs2404715	1	57008778	T	C	0.0925	1.8E-05	2.0E-02	-2.0E-01	8.0E-02	1.2E-02
rs2467099	17	73949045	T	C	0.2204	3.4E-05	3.7E-02	-1.9E-01	5.5E-02	4.8E-04
rs2493292	1	3328659	T	C	0.1501	4.9E-05	5.4E-02	2.7E-01	6.8E-02	7.8E-05
rs2521501	15	91437388	T	A	0.3301	3.7E-04	4.0E-01	5.6E-01	5.2E-02	4.8E-27
rs2579519	2	96675166	C	T	0.3949	3.7E-08	4.1E-05	5.4E-03	4.8E-02	9.1E-01
rs2645466	17	57853214	C	A	0.3018	4.4E-05	4.8E-02	2.0E-01	4.9E-02	7.1E-05
rs2759308	15	81016227	A	G	0.4758	9.0E-05	9.8E-02	2.6E-01	4.6E-02	1.8E-08
rs2761436	1	207919748	T	C	0.5276	5.0E-05	5.4E-02	1.9E-01	4.6E-02	2.9E-05
rs2782980	10	115781527	C	T	0.7096	1.3E-04	1.4E-01	3.4E-01	5.2E-02	7.6E-11

SNP	Chromosome number	Chromosome position	Effect allele	Other allele	Effect allele frequency	R-squared	F-statistic	β_{xj}	β_{xj} Standard error	β_{xj} P-value
rs28427409	17	6473882	T	C	0.4133	3.5E-05	3.8E-02	1.6E-01	4.6E-02	4.0E-04
rs2898290	8	11433909	C	T	0.5165	1.6E-04	1.7E-01	-3.4E-01	4.7E-02	2.1E-13
rs2932538	1	113216543	G	A	0.7414	1.2E-04	1.3E-01	3.4E-01	5.3E-02	7.2E-11
rs2969070	7	2512545	A	G	0.6369	9.4E-05	1.0E-01	-2.8E-01	4.8E-02	8.4E-09
rs2972146	2	227100698	T	G	0.6362	7.7E-05	8.4E-02	2.5E-01	4.8E-02	1.8E-07
rs2978098	8	101676675	C	A	0.442	2.9E-05	3.2E-02	-1.5E-01	4.7E-02	1.7E-03
rs2978456	8	42324765	C	T	0.4442	1.5E-05	1.7E-02	1.1E-01	5.0E-02	3.2E-02
rs3184504	12	111884608	C	T	0.5244	4.5E-04	4.9E-01	-5.8E-01	4.7E-02	2.6E-35
rs33063	16	69640217	G	A	0.856	2.1E-05	2.3E-02	-1.8E-01	6.5E-02	6.0E-03
rs34591516	8	142367087	T	C	0.0533	8.5E-05	9.3E-02	5.6E-01	1.1E-01	1.5E-07
rs347591	3	11290122	T	G	0.6625	9.7E-05	1.1E-01	2.8E-01	4.9E-02	6.3E-09
rs34872471	10	114754071	C	T	0.2936	3.6E-05	3.9E-02	1.8E-01	5.1E-02	4.2E-04
rs35261357	16	75444572	T	C	0.5698	1.0E-04	1.1E-01	2.8E-01	4.7E-02	2.7E-09
rs35410524	6	96885405	T	C	0.1917	7.5E-05	8.2E-02	3.0E-01	5.9E-02	3.4E-07
rs35444	12	115552437	G	A	0.3921	1.4E-04	1.5E-01	-3.3E-01	4.7E-02	1.8E-12
rs35783704	8	105966258	A	G	0.1092	1.4E-04	1.6E-01	-5.2E-01	7.7E-02	1.5E-11
rs36010659	18	48283949	C	T	0.1391	2.4E-05	2.7E-02	-2.0E-01	6.6E-02	3.0E-03
rs36022378	3	49913705	C	T	0.1926	2.0E-05	2.2E-02	1.6E-01	6.0E-02	9.3E-03
rs3741378	11	65408937	T	C	0.1328	1.1E-04	1.2E-01	-4.2E-01	7.0E-02	2.2E-09
rs3771371	2	71627539	T	C	0.5658	4.3E-05	4.7E-02	-1.8E-01	4.6E-02	9.2E-05
rs381815	11	16902268	T	C	0.2705	1.4E-04	1.5E-01	3.6E-01	5.2E-02	1.5E-12
rs3820068	1	15798197	G	A	0.2023	9.8E-05	1.1E-01	-3.4E-01	6.0E-02	1.7E-08
rs3918226	7	150690176	T	C	0.0811	1.2E-04	1.3E-01	5.5E-01	9.1E-02	1.8E-09
rs409558	6	31708147	C	T	0.1712	1.5E-04	1.7E-01	-4.5E-01	6.4E-02	2.6E-12
rs419076	3	169100886	C	T	0.5265	2.1E-04	2.3E-01	-3.9E-01	4.6E-02	5.4E-18
rs4245739	1	204518842	A	C	0.7326	3.0E-05	3.3E-02	1.7E-01	5.3E-02	1.2E-03
rs4247374	19	7252756	T	C	0.1355	1.6E-04	1.8E-01	-5.1E-01	7.5E-02	1.8E-11
rs4292285	4	145271954	A	T	0.3994	2.1E-05	2.3E-02	-1.3E-01	4.7E-02	5.8E-03
rs4308	17	61559625	G	A	0.6178	9.4E-05	1.0E-01	-2.7E-01	4.8E-02	1.1E-08
rs4364717	9	21801530	G	A	0.4534	1.5E-05	1.6E-02	1.1E-01	4.6E-02	1.9E-02
rs4373814	10	18419972	C	G	0.441	8.4E-05	9.2E-02	2.5E-01	4.7E-02	6.2E-08
rs4387287	10	105677897	C	A	0.8262	2.9E-05	3.2E-02	-2.0E-01	6.2E-02	1.8E-03
rs4454254	8	141060027	A	G	0.6346	6.1E-05	6.7E-02	-2.2E-01	4.8E-02	3.2E-06
rs4494250	10	96563757	A	G	0.3611	8.1E-05	8.9E-02	2.6E-01	4.9E-02	1.3E-07
rs449789	6	159699125	G	C	0.8652	1.8E-05	1.9E-02	-1.7E-01	6.8E-02	1.3E-02
rs452036	14	23865885	A	G	0.3492	2.5E-05	2.8E-02	-1.4E-01	4.8E-02	2.7E-03
rs470113	22	40729614	G	A	0.188	5.0E-06	5.5E-03	7.8E-02	5.8E-02	1.8E-01
rs4728142	7	128573967	A	G	0.4383	6.1E-05	6.7E-02	-2.2E-01	4.7E-02	3.9E-06
rs4757391	11	16302939	T	C	0.8013	2.1E-04	2.3E-01	-4.9E-01	5.7E-02	8.3E-18
rs4823006	22	29451671	G	A	0.4384	2.6E-05	2.9E-02	-1.4E-01	4.6E-02	2.0E-03
rs4952611	2	40567743	T	C	0.5834	5.9E-05	6.5E-02	-2.1E-01	4.9E-02	1.2E-05
rs5219	11	17409572	C	T	0.6245	1.3E-04	1.4E-01	-3.2E-01	4.7E-02	1.1E-11
rs55701159	2	25139596	G	T	0.113	4.8E-05	5.3E-02	-3.0E-01	7.4E-02	5.3E-05
rs55780018	2	208526140	C	T	0.452	1.4E-04	1.6E-01	3.3E-01	4.9E-02	1.9E-11
rs57927100	17	75317300	G	C	0.259	1.2E-04	1.4E-01	-3.5E-01	5.4E-02	8.4E-11
rs6015450	20	57751117	G	A	0.1287	2.6E-04	2.8E-01	6.6E-01	6.9E-02	2.4E-21
rs60199046	1	59663341	G	A	0.2868	3.5E-05	3.8E-02	-1.8E-01	5.1E-02	4.3E-04
rs6031435	20	42797358	G	A	0.4612	6.9E-05	7.5E-02	2.3E-01	4.6E-02	6.7E-07
rs6060114	20	30169673	C	T	0.162	5.8E-05	6.3E-02	-2.8E-01	6.2E-02	5.9E-06
rs6081613	20	19465907	A	G	0.2722	2.0E-05	2.1E-02	1.4E-01	5.1E-02	7.3E-03
rs6095241	20	47308798	A	G	0.4363	4.7E-05	5.1E-02	-1.9E-01	4.5E-02	3.3E-05
rs6108168	20	8626271	A	C	0.2541	4.2E-05	4.6E-02	-2.0E-01	5.2E-02	8.8E-05
rs62011052	15	79156983	C	T	0.1497	1.9E-07	2.0E-04	1.7E-02	6.4E-02	8.0E-01
rs62104477	19	30294991	T	G	0.3313	9.0E-06	9.8E-03	8.7E-02	4.9E-02	7.4E-02
rs62270945	3	128201889	T	C	0.0302	3.9E-06	4.3E-03	1.6E-01	1.6E-01	3.2E-01
rs62524579	8	144060955	A	G	0.5307	4.4E-05	4.8E-02	-1.8E-01	5.3E-02	6.9E-04
rs6271	9	136522274	T	C	0.0725	6.8E-05	7.4E-02	-4.3E-01	1.0E-01	2.1E-05
rs633185	11	100593538	C	G	0.7096	2.7E-04	3.0E-01	5.0E-01	5.1E-02	9.8E-23
rs6429422	1	243472801	G	T	0.3289	2.4E-05	2.7E-02	1.4E-01	4.9E-02	3.6E-03
rs6487543	12	26438189	A	G	0.7655	3.0E-05	3.2E-02	1.8E-01	5.6E-02	1.9E-03

SNP	Chromosome number	Chromosome position	Effect allele	Other allele	Effect allele frequency	R-squared	F-statistic	β_{xj}	β_{xj} Standard error	β_{xj} P-value
rs6557876	8	25900675	T	C	0.2511	1.4E-04	1.5E-01	-3.7E-01	5.3E-02	6.0E-12
rs6595838	5	127868199	A	G	0.2891	6.2E-05	6.7E-02	2.4E-01	5.1E-02	3.1E-06
rs661348	11	1905292	C	T	0.4368	1.5E-04	1.7E-01	3.4E-01	5.0E-02	9.6E-12
rs6686889	1	25030470	T	C	0.2581	7.5E-06	8.2E-03	8.5E-02	5.3E-02	1.1E-01
rs66887589	4	120509279	C	T	0.4814	4.4E-05	4.8E-02	1.8E-01	4.6E-02	9.0E-05
rs67330701	11	69079707	T	C	0.0958	3.6E-05	3.9E-02	-2.8E-01	9.1E-02	2.4E-03
rs6783086	3	133959552	T	C	0.409	8.6E-05	9.4E-02	2.6E-01	4.7E-02	3.9E-08
rs6797587	3	48197614	G	A	0.6797	9.2E-05	1.0E-01	2.8E-01	5.0E-02	1.5E-08
rs6825911	4	111381638	T	C	0.787	5.1E-05	5.5E-02	-2.4E-01	5.8E-02	3.7E-05
rs687621	9	136137065	G	A	0.3392	1.2E-05	1.3E-02	-9.8E-02	4.8E-02	4.3E-02
rs6891344	5	123136656	G	A	0.1856	6.5E-05	7.1E-02	-2.8E-01	6.0E-02	2.2E-06
rs6911827	6	22130601	T	C	0.4623	3.1E-05	3.4E-02	1.5E-01	4.7E-02	1.3E-03
rs6969780	7	27159136	C	G	0.0961	6.4E-05	7.0E-02	3.7E-01	7.9E-02	3.1E-06
rs709209	1	6278414	G	A	0.3421	1.1E-05	1.2E-02	-9.4E-02	5.3E-02	7.6E-02
rs7103648	11	47461783	G	A	0.3844	1.2E-04	1.3E-01	3.1E-01	4.7E-02	6.2E-11
rs7126805	11	828916	A	G	0.7292	1.5E-05	1.7E-02	1.2E-01	5.6E-02	3.1E-02
rs7129220	11	10350538	A	G	0.1233	8.9E-05	9.7E-02	3.9E-01	7.2E-02	6.3E-08
rs7178615	15	66869072	G	A	0.6085	2.6E-05	2.8E-02	1.4E-01	4.7E-02	2.8E-03
rs7236548	18	43097750	A	C	0.1857	5.7E-05	6.2E-02	2.6E-01	5.8E-02	5.5E-06
rs7248104	19	7224431	A	G	0.4085	3.3E-05	3.6E-02	-1.6E-01	4.6E-02	6.0E-04
rs7255	2	20878820	C	T	0.5345	6.7E-06	7.3E-03	7.1E-02	4.7E-02	1.3E-01
rs72765298	9	127900996	C	T	0.1179	6.8E-05	7.4E-02	3.5E-01	7.3E-02	1.7E-06
rs72799341	16	30936743	A	G	0.2392	8.8E-06	9.6E-03	9.5E-02	5.4E-02	7.9E-02
rs72812846	5	173377636	A	T	0.278	6.1E-05	6.7E-02	-2.4E-01	5.3E-02	8.2E-06
rs7297416	12	54443090	C	A	0.3133	9.2E-05	1.0E-01	-2.8E-01	5.0E-02	1.8E-08
rs7302981	12	50537815	G	A	0.6131	1.8E-04	2.0E-01	-3.7E-01	4.6E-02	4.4E-16
rs73030266	6	166179459	T	A	0.0674	3.3E-05	3.6E-02	-3.1E-01	9.8E-02	1.4E-03
rs73091767	1	227250775	C	T	0.2654	8.6E-06	9.4E-03	9.1E-02	5.2E-02	7.9E-02
rs73099903	12	53440779	T	C	0.0794	7.0E-05	7.6E-02	4.2E-01	8.8E-02	1.6E-06
rs73161324	22	42038786	T	C	0.0591	1.3E-05	1.5E-02	2.1E-01	1.2E-01	6.7E-02
rs740406	19	2232221	G	A	0.0631	9.6E-05	1.0E-01	5.5E-01	1.0E-01	6.9E-08
rs7406910	17	46688256	C	T	0.9107	1.0E-04	1.1E-01	4.9E-01	8.1E-02	1.9E-09
rs740698	17	60767151	T	C	0.5726	2.8E-05	3.0E-02	-1.5E-01	4.8E-02	2.2E-03
rs745821	18	48142854	G	T	0.2509	3.2E-05	3.5E-02	-1.8E-01	5.3E-02	7.5E-04
rs7480089	11	45207851	A	G	0.1222	3.1E-05	3.4E-02	-2.3E-01	7.2E-02	1.1E-03
rs7500448	16	83045790	G	A	0.2531	5.1E-05	5.6E-02	-2.3E-01	5.3E-02	2.4E-05
rs7515635	1	42408070	C	T	0.5316	7.6E-05	8.3E-02	-2.4E-01	4.6E-02	2.7E-07
rs751984	11	61278246	C	T	0.1211	1.1E-04	1.2E-01	-4.3E-01	7.4E-02	4.1E-09
rs7562	2	28635740	C	T	0.4703	3.2E-05	3.5E-02	-1.6E-01	4.7E-02	9.3E-04
rs7592578	2	191439591	G	T	0.7926	1.0E-04	1.1E-01	3.4E-01	6.0E-02	1.5E-08
rs76206723	7	40447971	A	G	0.1097	5.9E-05	6.4E-02	-3.4E-01	7.4E-02	6.4E-06
rs76326501	2	43167878	C	A	0.0891	1.3E-04	1.4E-01	-5.5E-01	8.3E-02	3.1E-11
rs76452347	9	35906471	T	C	0.2058	2.6E-05	2.8E-02	-1.7E-01	6.2E-02	5.8E-03
rs76785029	12	94882905	T	C	0.0717	2.2E-06	2.5E-03	-7.9E-02	9.7E-02	4.2E-01
rs7777128	7	27337113	C	G	0.0816	1.2E-04	1.3E-01	5.5E-01	8.4E-02	4.7E-11
rs7810028	7	139461616	G	C	0.1945	4.1E-05	4.5E-02	-2.2E-01	5.8E-02	1.5E-04
rs78378222	17	7571752	G	T	0.0164	2.9E-06	3.2E-03	-1.8E-01	2.1E-01	3.8E-01
rs78648104	6	50683009	C	T	0.1015	6.2E-05	6.8E-02	3.6E-01	8.3E-02	1.7E-05
rs79089478	17	40317241	C	T	0.0278	2.8E-06	3.1E-03	-1.4E-01	1.5E-01	3.4E-01
rs7914287	10	69350563	C	T	0.2303	2.1E-05	2.3E-02	1.5E-01	5.6E-02	7.8E-03
rs79146658	2	179786068	C	T	0.0821	8.2E-09	8.9E-06	4.5E-03	8.6E-02	9.6E-01
rs7927515	11	76125330	A	C	0.3455	3.5E-05	3.9E-02	1.7E-01	4.9E-02	4.8E-04
rs7977389	12	49981722	C	T	0.1065	1.4E-05	1.5E-02	-1.6E-01	7.5E-02	2.8E-02
rs8059962	16	81574197	C	T	0.5764	3.7E-05	4.1E-02	1.7E-01	4.7E-02	3.0E-04
rs8105753	19	31927547	C	A	0.3745	4.5E-05	4.9E-02	-1.9E-01	4.9E-02	9.9E-05
rs8258	11	117283676	C	T	0.6324	3.9E-06	4.3E-03	-5.6E-02	4.8E-02	2.4E-01
rs869396	4	169688000	A	C	0.4668	4.3E-05	4.7E-02	-1.8E-01	4.7E-02	1.1E-04
rs871606	4	54799245	C	T	0.1058	6.6E-05	7.2E-02	-3.6E-01	7.6E-02	2.2E-06
rs880315	1	10796866	C	T	0.348	3.3E-04	3.6E-01	5.2E-01	5.0E-02	1.3E-25
rs8904	14	35871217	A	G	0.3741	1.3E-04	1.4E-01	3.2E-01	4.8E-02	2.0E-11

SNP	Chromosome number	Chromosome position	Effect allele	Other allele	Effect allele frequency	R-squared	F-statistic	β_{xj}	β_{xj} Standard error	β_{xj} P-value
rs891511	7	150704843	A	G	0.3518	7.8E-05	8.5E-02	-2.5E-01	5.2E-02	1.2E-06
rs894344	8	135612745	G	A	0.4105	2.8E-05	3.0E-02	1.5E-01	4.7E-02	1.8E-03
rs900145	11	13293905	T	C	0.7041	1.4E-05	1.6E-02	1.1E-01	5.0E-02	2.3E-02
rs917275	7	28658522	G	A	0.3892	1.6E-05	1.8E-02	1.1E-01	4.8E-02	1.8E-02
rs918466	3	64710253	A	G	0.4105	2.5E-05	2.7E-02	-1.4E-01	4.8E-02	3.6E-03
rs9306160	21	45107562	C	T	0.6026	5.5E-05	6.0E-02	2.1E-01	4.7E-02	1.3E-05
rs9323988	14	98587630	C	T	0.3896	7.2E-05	7.9E-02	2.4E-01	4.6E-02	3.1E-07
rs932764	10	95895940	G	A	0.4439	1.8E-04	1.9E-01	3.7E-01	4.7E-02	4.8E-15
rs9337951	10	30317073	A	G	0.337	1.0E-05	1.1E-02	9.3E-02	5.3E-02	7.8E-02
rs9349379	6	12903957	G	A	0.4088	8.9E-05	9.8E-02	-2.6E-01	4.9E-02	6.4E-08
rs9372498	6	118572486	A	T	0.0848	5.4E-05	5.9E-02	3.6E-01	8.3E-02	1.4E-05
rs9479200	6	152398505	G	A	0.1231	5.6E-06	6.1E-03	9.9E-02	7.1E-02	1.7E-01
rs9549328	13	113636156	T	C	0.2345	4.6E-05	5.0E-02	2.2E-01	5.5E-02	8.6E-05
rs956006	15	62808539	T	C	0.336	5.8E-06	6.4E-03	-7.0E-02	4.9E-02	1.6E-01
rs9662255	1	9441949	A	C	0.4307	4.7E-05	5.1E-02	-1.9E-01	4.8E-02	9.4E-05
rs9678851	2	27887034	A	C	0.559	1.7E-05	1.9E-02	-1.1E-01	4.7E-02	1.7E-02
rs9687065	5	148391140	G	A	0.1957	5.3E-05	5.8E-02	-2.5E-01	5.9E-02	2.3E-05
rs9729719	1	38298207	A	G	0.2946	1.1E-05	1.3E-02	1.0E-01	5.3E-02	5.6E-02
rs9810888	3	53635595	G	T	0.5024	3.0E-05	3.3E-02	1.5E-01	4.6E-02	1.2E-03
rs9815354	3	41912651	A	G	0.1711	3.0E-06	3.3E-03	-6.3E-02	6.3E-02	3.2E-01
rs9827472	3	56726646	T	C	0.3577	3.3E-05	3.6E-02	-1.6E-01	4.9E-02	7.2E-04
rs9888615	14	53377540	C	T	0.7064	6.2E-05	6.7E-02	2.4E-01	5.0E-02	2.3E-06

Table S3. UK prevalence estimates used to calculate population impact of distributional shifts in blood pressure.

Outcome	UK prevalence (per 10,000)	Age group (years)	Year	Source of prevalence estimate
**Coronary Artery Disease**	612	45-64	2017	Health Survey for England [1]
**Ischemic Cerebrovascular Disease (All)**	280	45-64	2017	Health Survey for England [1]
**Hemorrhagic Stroke (All)**	84.7	50-69	2010	Global Burden of Disease [2]
**Peripheral Vascular Disease**	240	50-89	2014	The Health Improvement Network [3]
**Aortic Valve Stenosis**	96.0	45-64	2017	Health Survey for England [1]
**Atrial Fibrillation**	250	45-69	2017	Public Health England [4]
**Heart failure**	80.0	45-74	2017	Quality and Outcomes Framework [5]
**Dilated Cardiomyopathy**	20.0	45-75	2014	British Heart Foundation [6]
**Endocarditis**	11	All ages[a]	2013	Hospital Episode Statistics [7]
**Rheumatic Heart Disease**	23.0	All ages[a]	2013	Hospital Episode Statistics [8]
**Chronic Kidney Disease**	700	45-64	2017	Health Survey for England [1]

[a] When no age group-specific estimates could be obtained, the total population prevalence was used.

1. NHS Digital. Health Survey for England 2017. https://digital.nhs.uk/data-and-information/publications/statistical/health-survey-for-england/2017.

2. Global Burden of Disease Collaborative Network. Global Burden of Disease Study 2017 (GBD 2017) Results. http://ghdx.healthdata.org/gbd-results-tool. Published 2018.

3. Cea-Soriano L, Fowkes FGR, Johansson S, Allum AM, García Rodriguez LA. Time trends in peripheral artery disease incidence, prevalence and secondary preventive therapy: a cohort study in The Health Improvement Network in the UK. *BMJ Open*. 2018;8(1):e018184. doi:10.1136/bmjopen-2017-018184

4. Public Health England. Atrial fibrillation prevalence estimates in England : Application of recent population estimates of AF. *PHE Publ Gateway number 2014778*. 2015;August:1-4.

5. NHS Digital. Quality and Outcomes Framework, Achievement, prevalence and exceptions data - 2017-18. https://digital.nhs.uk/data-and-information/publications/statistical/quality-and-outcomes-framework-achievement-prevalence-and-exceptions-data/2017-18.

6. Cardiomyopathy - British Heart Foundation. https://www.bhf.org.uk/informationsupport/conditions/cardiomyopathy.

7. Dayer MJ, Jones S, Prendergast B, Baddour LM, Lockhart PB, Thornhill MH. Incidence of infective endocarditis in England, 2000-13: a secular trend, interrupted time-series analysis. *Lancet*. 2015;385(9974):1219-1228. doi:10.1016/S0140-6736(14)62007-9

8. Bhatnagar P, Wickramasinghe K, Williams J, Rayner M, Townsend N. The epidemiology of cardiovascular disease in the UK 2014. *Heart*. 2015;101(15):1182-1189. doi:10.1136/heartjnl-2015-307516.

Table S4. Mendelian randomization estimates (odds ratio with 95% confidence interval per 10 mmHg increase in genetically-predicted systolic blood pressure) using the inverse-variance weighted, MR-Egger, weighted median, and MR-PRESSO methods for 21 disease outcomes.

Outcome	Inverse-weighted variance		MR-Egger		Weighted median		MR-PRESSO	
	OR (95% CI)	P-value	OR (95% CI)	P-value	OR (95% CI)	P-value	OR (95% CI)	P-value
Coronary Artery Disease	1.59 (1.45-1.74)	<0.001	1.44 (1.20-1.73)	<0.001	1.61 (1.48-1.75)	<0.001	1.59 (1.45-1.75)	<0.001
Ischemic Cerebrovascular Disease (all)	1.52 (1.39-1.66)	<0.001	1.66 (1.40-1.98)	<0.001	1.50 (1.33-1.70)	<0.001	1.52 (1.39-1.66)	<0.001
Ischemic Stroke	1.70 (1.52-1.90)	<0.001	1.86 (1.48-2.32)	<0.001	1.78 (1.51-2.09)	<0.001	1.71 (1.52-1.91)	<0.001
Transient Ischemic Attack	1.34 (1.19-1.51)	<0.001	1.40 (1.10-1.79)	0.006	1.38 (1.15-1.64)	<0.001	1.35 (1.19-1.52)	<0.001
Hemorrhagic Stroke (all)	1.43 (1.21-1.69)	<0.001	1.39 (0.99-1.95)	0.05	1.33 (1.04-1.71)	0.02	1.42 (1.20-1.68)	<0.001
Intracerebral Hemorrhage	1.32 (1.06-1.66)	0.02	1.22 (0.78-1.91)	0.39	1.24 (0.88-1.74)	0.21	1.31 (1.04-1.64)	0.02
Subarachnoid Hemorrhage	1.54 (1.22-1.94)	<0.001	1.72 (1.09-2.72)	0.02	1.65 (1.17-2.32)	0.004	1.53 (1.22-1.94)	<0.001
Aortic Aneurysm (all)	1.26 (1.02-1.56)	0.03	1.51 (0.99-2.31)	0.06	1.39 (1.06-1.82)	0.02	1.28 (1.03-1.58)	0.02
Abdominal Aortic Aneurysm	1.20 (0.93-1.56)	0.17	1.07 (0.64-1.79)	0.80	1.34 (0.95-1.88)	0.10	1.22 (0.94-1.58)	0.15
Thoracic Aortic Aneurysm	1.19 (0.79-1.77)	0.41	1.99 (0.90-4.41)	0.09	1.34 (0.75-2.40)	0.33	1.23 (0.82-1.84)	0.31
Venous Thromboembolism (all)	0.90 (0.80-1.01)	0.07	1.00 (0.79-1.25)	0.98	0.87 (0.78-0.96)	0.005	0.90 (0.80-1.01)	0.07
Deep Vein Thrombosis	0.88 (0.77-1.00)	0.04	0.95 (0.74-1.22)	0.67	0.90 (0.80-1.02)	0.09	0.87 (0.77-0.99)	0.04
Pulmonary Embolism	0.91 (0.79-1.05)	0.20	1.05 (0.79-1.38)	0.75	0.96 (0.83-1.11)	0.57	0.92 (0.80-1.05)	0.22
Peripheral Vascular Disease	1.28 (1.11-1.46)	<0.001	1.01 (0.77-1.32)	0.96	1.18 (0.98-1.43)	0.09	1.28 (1.11-1.47)	<0.001
Aortic Valve Stenosis	1.74 (1.48-2.04)	<0.001	1.90 (1.38-2.62)	<0.001	1.81 (1.43-2.29)	<0.001	1.75 (1.49-2.05)	<0.001
Atrial Fibrillation	1.32 (1.21-1.42)	<0.001	1.35 (1.15-1.58)	<0.001	1.29 (1.17-1.42)	<0.001	1.32 (1.21-1.43)	<0.001
Heart Failure	1.38 (1.25-1.53)	<0.001	1.36 (1.12-1.67)	0.002	1.47 (1.28-1.69)	<0.001	1.37 (1.24-1.51)	<0.001
Dilated Cardiomyopathy	1.61 (1.24-2.10)	<0.001	2.57 (1.52-4.35)	<0.001	1.60 (1.07-2.40)	0.02	1.58 (1.23-2.03)	<0.001
Endocarditis	1.49 (1.12-1.99)	0.007	1.41 (0.79-2.52)	0.24	1.47 (0.97-2.24)	0.07	1.52 (1.14-2.03)	<0.001
Rheumatic Heart Disease	1.32 (1.13-1.53)	<0.001	1.55 (1.15-2.09)	0.004	1.43 (1.16-1.77)	0.001	1.31 (1.13-1.52)	<0.001
Chronic Kidney Disease	1.39 (1.24-1.55)	<0.001	1.17 (0.95-1.46)	0.14	1.38 (1.19-1.59)	<0.001	1.39 (1.24-1.55)	<0.001

Table S5. Population impact fractions (PIFs) for outcomes with strong evidence of causality for systolic blood pressure. The PIF represents the percentage reduction (with 95% confidence interval) in events if systolic blood pressure was 132.7 mmHg, 127.7 mmHg, and 115.0 mmHg for all individuals in the UK Biobank study sample, instead of the current mean systolic blood pressure of 137.7 mmHg.

Outcomes	Percentage reduction in number of events attributable to setting systolic blood pressure to:		
	**132.7 mmHg (-5 mmHg)**	**127.7 mmHg (-10 mmHg)**	**115.0 mmHg (-22.7 mmHg)**
**Coronary Artery Disease**	20.9 (17.3-24.4)	37.5 (31.6-42.9)	65.6 (57.8-72.0)
**Ischemic Cerebrovascular Disease (all)[a]**	18.9 (15.2-22.5)	34.3 (28.1-40.0)	61.5 (52.7-68.6)
**Hemorrhagic Stroke (all)[a]**	16.1 (8.6-22.9)	29.5 (16.5-40.5)	54.8 (33.5-69.3)
**Peripheral Vascular Disease**	11.8 (5.4-17.7)	22.1 (10.4-32.3)	43.3 (22.1-58.7)
**Aortic Valve Stenosis**	24.4 (18.1-30.2)	42.9 (33.0-51.3)	72.0 (59.7-80.5)
**Atrial Fibrillation**	12.6 (9.1-16.5)	23.7 (17.3-30.2)	45.8 (35.0-55.8)
**Heart Failure**	14.4 (10.0-18.5)	26.7 (18.9-33.6)	50.5 (37.9-60.6)
**Dilated Cardiomyopathy**	20.5 (9.1-30.2)	36.9 (17.3-51.3)	64.8 (35.0-80.5)
**Endocarditis**	18.9 (6.3-29.9)	34.2 (12.2-50.8)	61.4 (25.6-80.0)
**Rheumatic Heart Disease**	12.6 (5.8-18.9)	23.7 (11.3-34.3)	45.8 (23.9-61.5)
**Chronic Kidney Disease**	15.2 (10.4-19.8)	28.1 (19.8-35.7)	52.7 (39.3-63.2)
**Total**	**16.9 (12.2-21.3)**	**30.8 (22.8-38.0)**	**56.2 (43.7-65.9)**

[a] To minimize double counting, we present single estimates for both ischemic cerebrovascular disease and hemorrhagic stroke

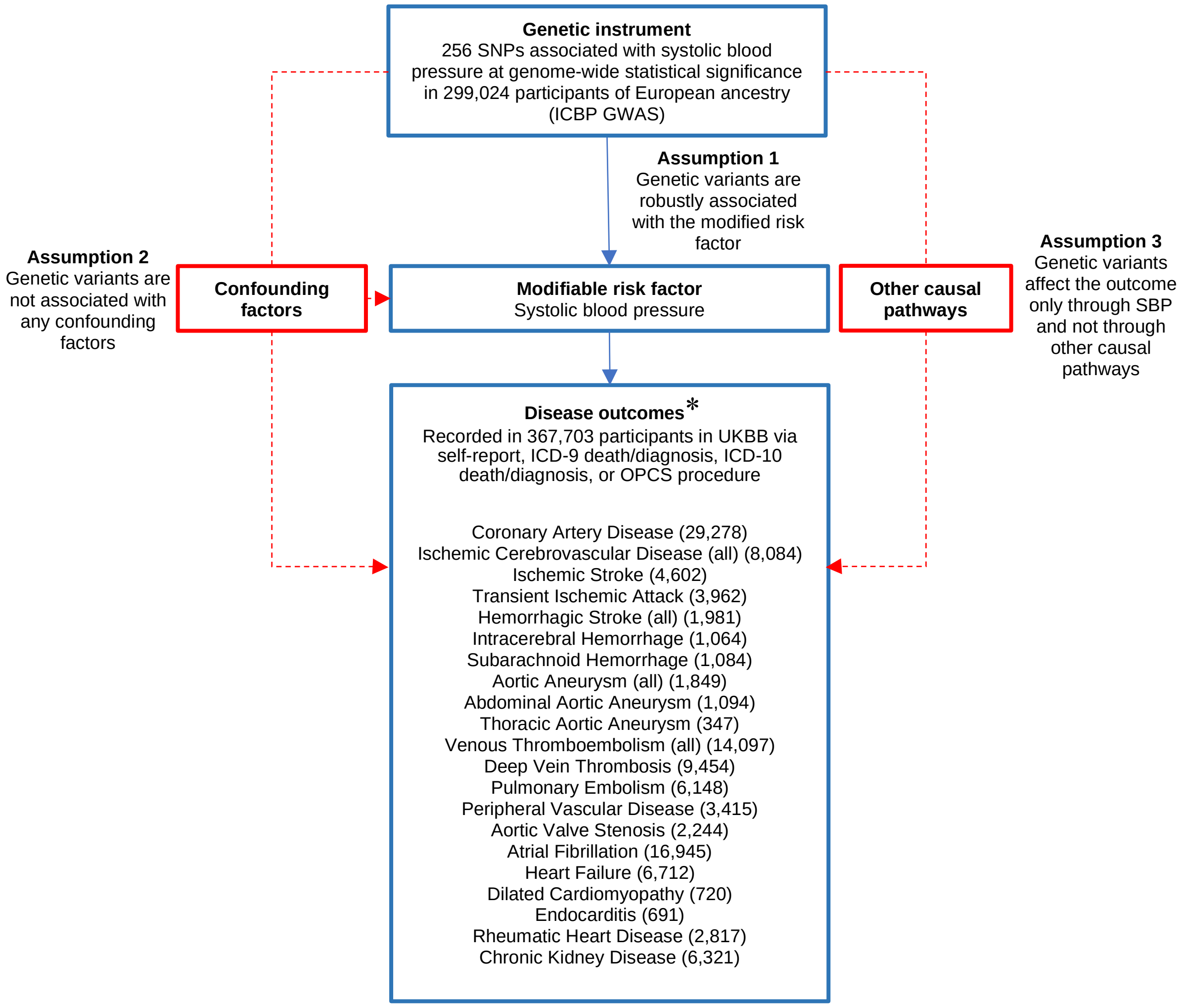

Figure S1. Summary of the data sources for this study and the assumptions of the Mendelian randomization design. Broken lines represent potential pleiotropic or direct causal effects between variables that would violate the Mendelian randomization assumptions.

*The number of cases for each outcome is reported in parentheses.

GWAS, Genome-wide association study; ICBP, International Blood Pressure Consortium; ICD, International Classification of Disease; OPCS, Office of Population Censuses and Surveys Classification of Surgical Operations and Procedures; SBP, systolic blood pressure; SNP, single nucleotide polymorphism; UKBB, UK Biobank.

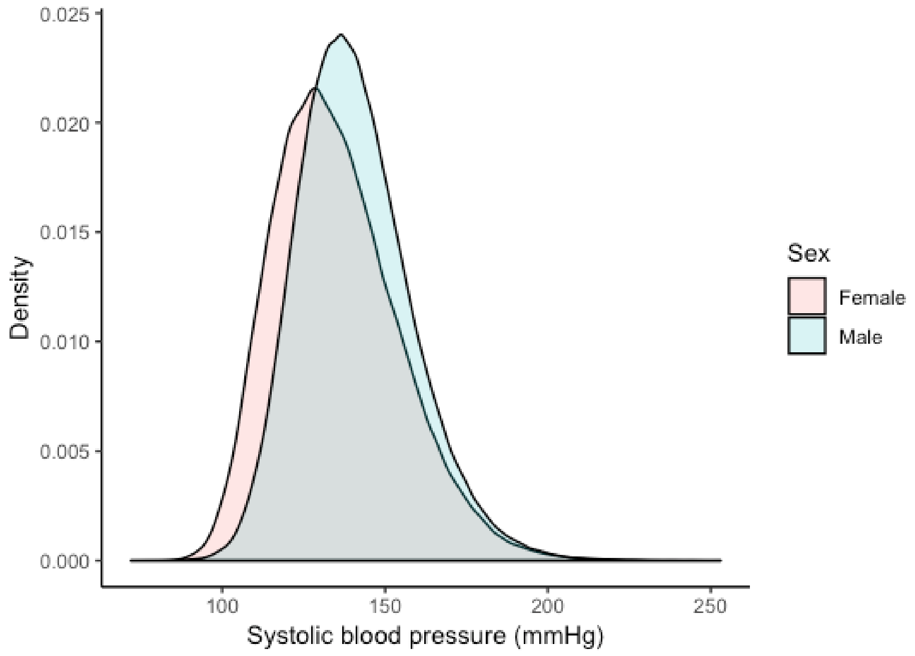

Figure S2. Distribution of systolic blood pressure in UK Biobank. Overall mean systolic blood pressure is 137.7 mmHg (standard deviation 18.6 mmHg); female mean systolic blood pressure is 135.0 mmHg (standard deviation 19.2); male mean systolic blood pressure is 140.8 (standard deviation is 17.4 mmHg).

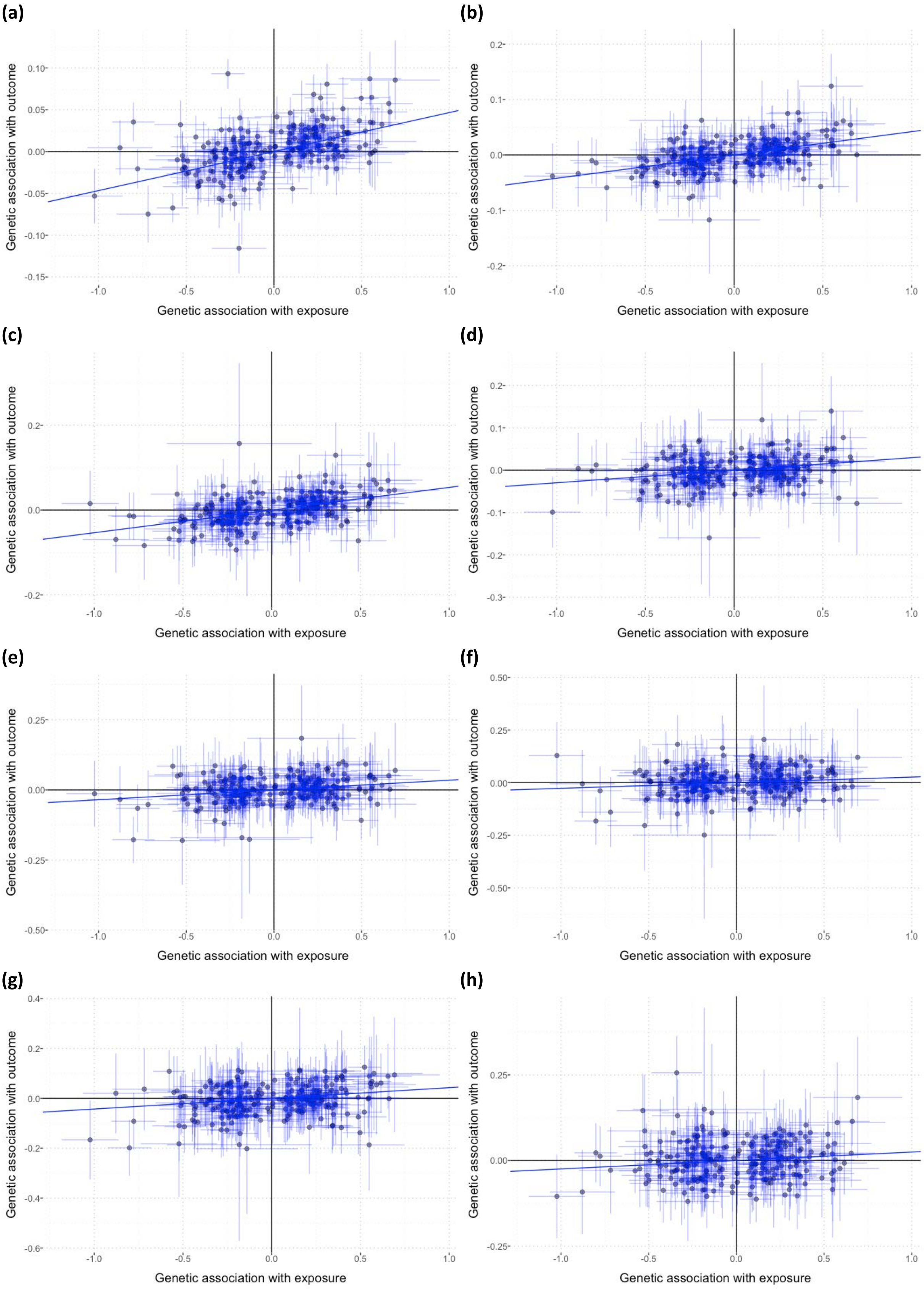

Figure S3 (a)-(m). Scatter plots of the beta-coefficients for the exposure plotted against the beta-coefficients for the outcome for each of the 256 SNPs in the instrument. Each dot corresponds to a SNP. Lines represent the 95% CI for each SNP's beta-coefficient. Each scatter plot corresponds to one outcome: (a) Coronary artery disease; (b) Ischemic cerebrovascular disease (all); (c) Ischemic stroke; (d) Transient ischemic attack; (e) Hemorrhagic stroke (all); (f) Intracerebral hemorrhage; (g) Subarachnoid hemorrhage; (h) Aortic aneurysm (all); (i) Abdominal aortic aneurysm; (j) Thoracic aortic aneurysm; (k) Venous thromboembolism (all); (l) Deep vein thrombosis; (m) Pulmonary embolism; (n) Peripheral vascular disease; (o) Aortic valve stenosis; (p) Atrial fibrillation; (q) Heart failure; (r) Dilated cardiomyopathy; (s) Endocarditis; (t) Rheumatic heart disease; (u) Chronic kidney disease.

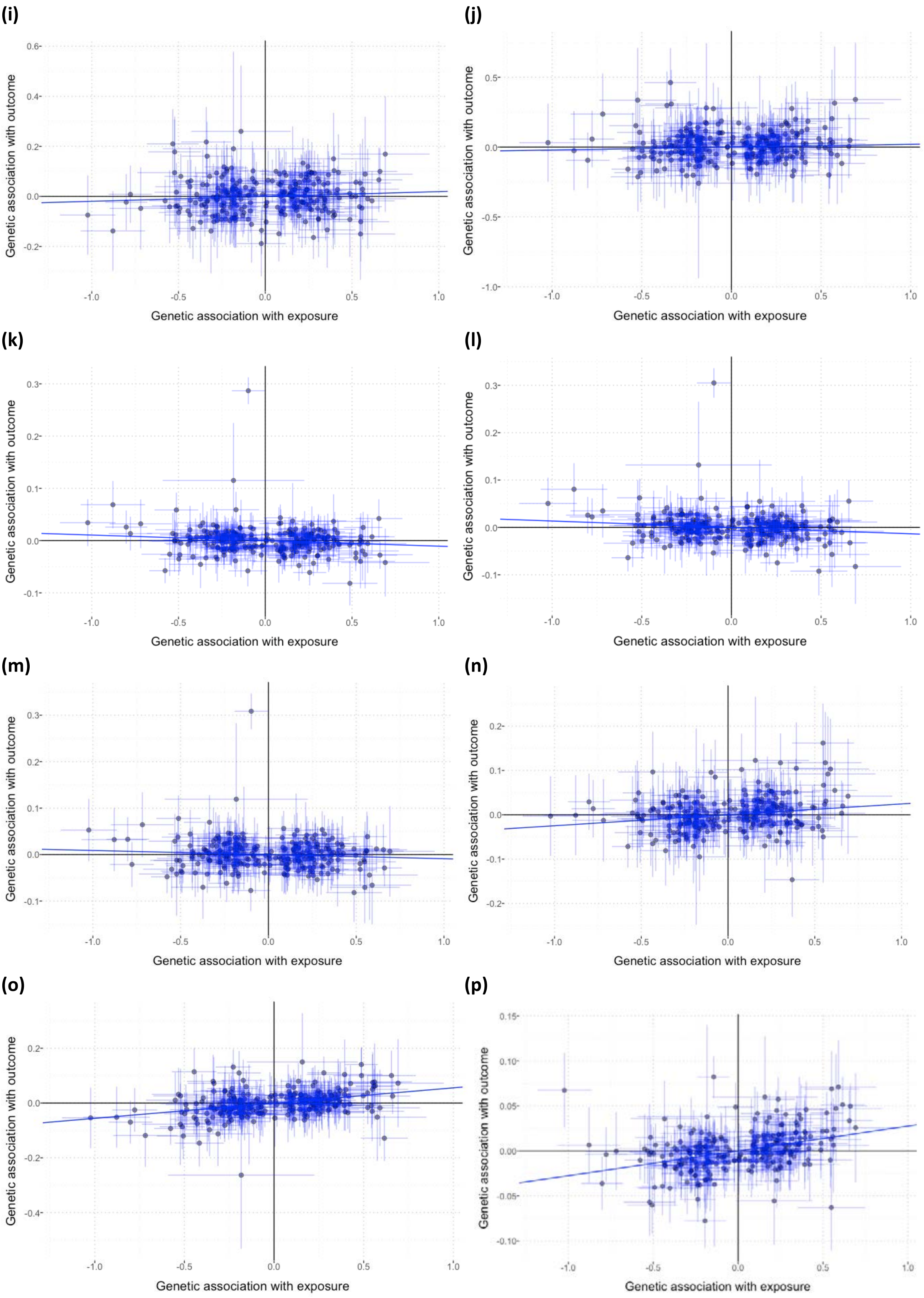

Figure S3 (contd.) (a)-(m). Scatter plots of the beta-coefficients for the exposure plotted against the beta-coefficients for the outcome for each of the 256 SNPs in the instrument. Each dot corresponds to a SNP. Lines represent the 95% CI for each SNP's beta-coefficient. Each scatter plot corresponds to one outcome: (a) Coronary artery disease; (b) Ischemic cerebrovascular disease (all); (c) Ischemic stroke; (d) Transient ischemic attack; (e) Hemorrhagic stroke (all); (f) Intracerebral hemorrhage; (g) Subarachnoid hemorrhage; (h) Aortic aneurysm (all); (i) Abdominal aortic aneurysm; (j) Thoracic aortic aneurysm; (k) Venous thromboembolism (all); (l) Deep vein thrombosis; (m) Pulmonary embolism; (n) Peripheral vascular disease; (o) Aortic valve stenosis; (p) Atrial fibrillation; (q) Heart failure; (r) Dilated cardiomyopathy; (s) Endocarditis; (t) Rheumatic heart disease; (u) Chronic kidney disease.

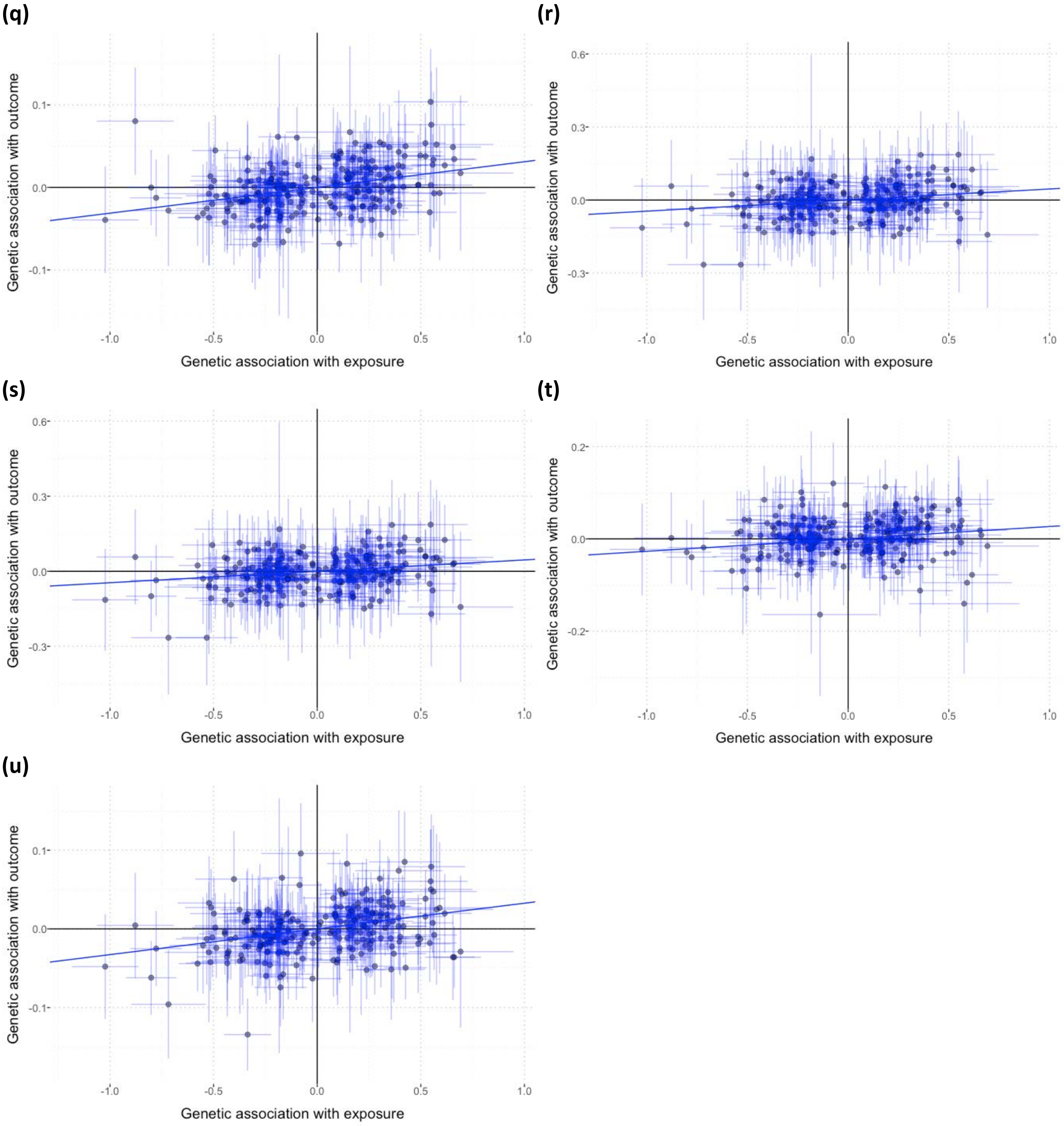

Figure S3 (contd.) (a)-(m). Scatter plots of the beta-coefficients for the exposure plotted against the beta-coefficients for the outcome for each of the 256 SNPs in the instrument. Each dot corresponds to a SNP. Lines represent the 95% CI for each SNP's beta-coefficient. Each scatter plot corresponds to one outcome: (a) Coronary artery disease; (b) Ischemic cerebrovascular disease (all); (c) Ischemic stroke; (d) Transient ischemic attack; (e) Hemorrhagic stroke (all); (f) Intracerebral hemorrhage; (g) Subarachnoid hemorrhage; (h) Aortic aneurysm (all); (i) Abdominal aortic aneurysm; (j) Thoracic aortic aneurysm; (k) Venous thromboembolism (all); (l) Deep vein thrombosis; (m) Pulmonary embolism; (n) Peripheral vascular disease; (o) Aortic valve stenosis; (p) Atrial fibrillation; (q) Heart failure; (r) Dilated cardiomyopathy; (s) Endocarditis; (t) Rheumatic heart disease; (u) Chronic kidney disease.
